# Krüppel-like factor 2: a central regulator of B cell differentiation and plasma cell homing

**DOI:** 10.3389/fimmu.2023.1172641

**Published:** 2023-05-12

**Authors:** Jens Wittner, Wolfgang Schuh

**Affiliations:** Division of Molecular Immunology, Department of Internal Medicine 3, Nikolaus-Fiebiger-Center, University Hospital Erlangen, Friedrich-Alexander-Universität Erlangen-Nürnberg, Erlangen, Germany

**Keywords:** KLF2, plasma cells, quiescence, B cells, integrins, IgA, mucosal immunity, multiple myeloma

## Abstract

The development of B cells, their activation and terminal differentiation into antibody-producing plasma cells are characterized by alternating phases of proliferation and quiescence that are controlled by complex transcriptional networks. The spatial and anatomical organization of B cells and plasma cells inside lymphoid organs as well as their migration within lymphoid structures and between organs are prerequisites for the generation and the maintenance of humoral immune responses. Transcription factors of the Krüppel-like family are critical regulators of immune cell differentiation, activation, and migration. Here, we discuss the functional relevance of Krüppel-like factor 2 (KLF2) for B cell development, B cell activation, plasma cell formation and maintenance. We elaborate on KLF2-mediated regulation of B cell and plasmablast migration in the context of immune responses. Moreover, we describe the importance of KLF2 for the onset and the progression of B cell-related diseases and malignancies.

## Introduction

Krüppel-like factor 2 (KLF2) is a transcription factor of the Krüppel-like factor (KLF) family whose members are characterized by a C-terminal zinc finger DNA-binding domain. The family name originated from the phenotype of a Drosophila loss-of-function mutant with abnormal segmentation of the abdominal region of the Drosophila larva (“Krüppel” mutant, Krüppel: German word for cripple). In Drosophila, the *krüppel* gene is one of the so-called *gap* genes, a group of genes responsible for the development of the Drosophila larvae and their segmentation (1, 2). The KLF family consists of 17 members in vertebrates, all of which are involved in the control of differentiation, proliferation, cell adhesion, and migration processes in a variety of cell types (3, 4). KLF2 was first described by Anderson and colleagues in 1995 and originally named lung Krüppel-like factor (LKLF) due to its high expression in the lung (5). The importance of KLF2 during embryonic development was revealed in 1997 by Kuo and colleagues using a genomic knockout mouse model for the *Klf2* gene. Their study demonstrated that KLF2-deficient embryos died between days E12.5 and E14.5 due to hemorrhage, defective blood vessels, and an abnormal tunica media *in utero* (6). Thus, KLF2 has an essential function in embryonic development and in endothelial cell biology. From the time point of its discovery in the late 1990s, numerous studies have revealed a crucial role for KLF2 during proliferation, differentiation, activation, and positioning of B and T cells, and other immune cells (4, 7). The loss of function of KLF2 is associated with diseases, such as arteriosclerosis, adipogenesis, thrombosis, and lymphoma (3, 4, 7–12). The role of KLF2 has been intensively studied in T-lymphoid cells and it becomes increasingly evident that KLF2 also acts as an important regulator of different aspects of B cell biology. Therefore, in this review article, we discuss the relevance of KLF2 during B cell differentiation and activation as well as its function of KLF2 as a regulator of B cell and plasma cell homing. Finally, we elaborate on how KLF2 contributes to B cell-related diseases and malignancies.

## Expression of KLF2 in B-lymphoid cells

**Figure 1:**
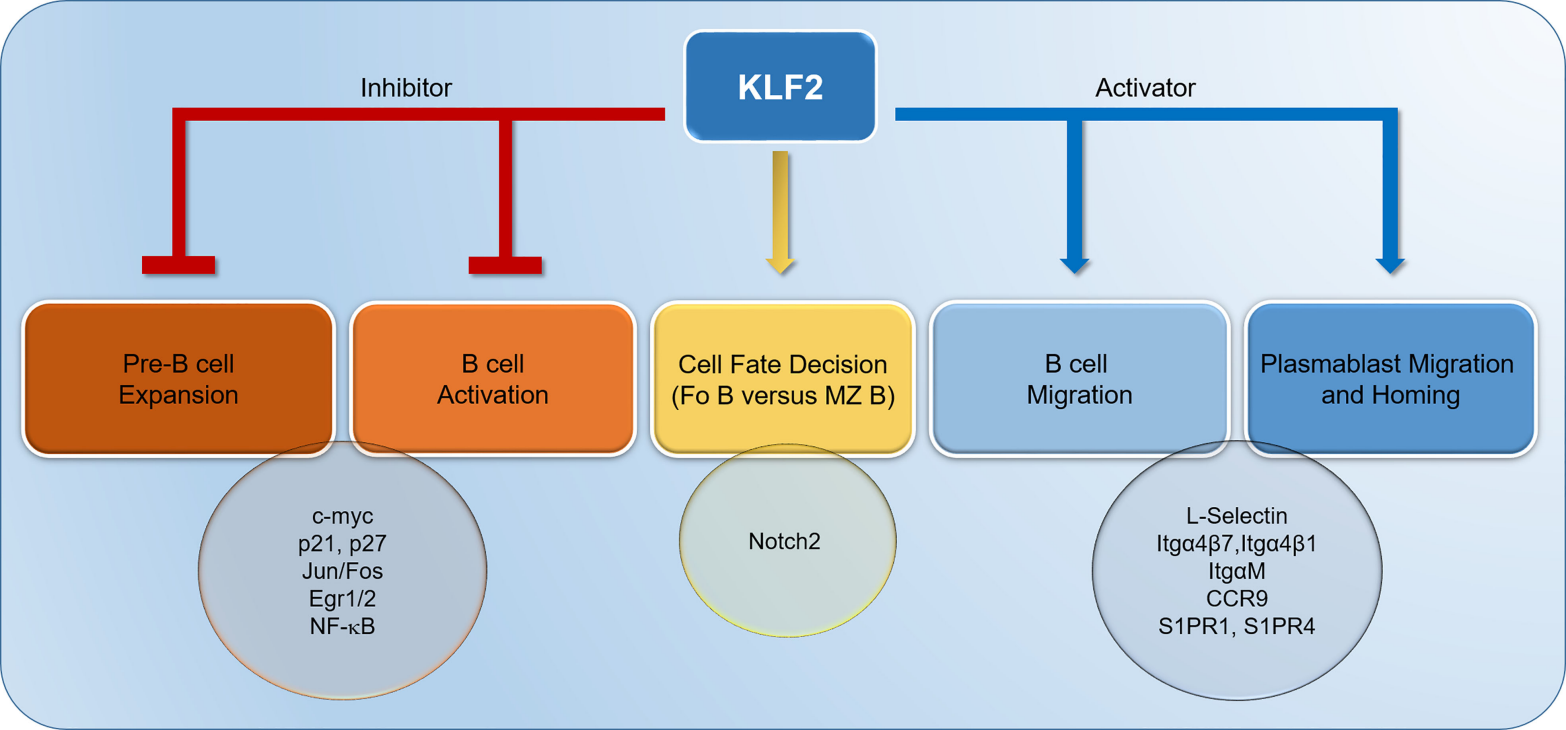
Krüppel-like factor-2 (KLF2) contributes to the termination of pre-B cell expansion through inhibition of Jun/Fos and Egr1/2. Moreover, KLF2 inhibits the proliferation of pre-B cells and the activation of naïve, mature B cells by downregulating c-myc and upregulating p21 and p27. In B cells, KLF2 suppresses NF-κB activation. Furthermore, KLF2 represses Notch2 signaling in naïve B cells, thereby driving B cell differentiation to follicular B cells. KLF2 controls the migration of B cells and plasmablasts by positively regulating L-Selectin, Itgα4β7, Itgα4β1, ItgαM, Chemokine receptor 9 (CCR9), Sphingosine-1-Phosphate-Receptor (S1PR) 1 and S1PR4. IgA plasmablast homing to gut-associated lymphoid tissues (GALT) is mediated by KLF2-regulated factors Itgα4β7 and CCR9. Itg, integrin.

Expression of KLF2 in early B cell progenitors in the bone marrow (BM) was discovered in a mouse model with tetracycline-controllable expression of the pre-B cell receptor (pre-BCR) (13). The pre-BCR is part of a critical checkpoint in early B cell development, which tests the ability of newly formed immunoglobulin (Ig) µ-heavy chains (µHC) to functionally pair with the surrogate light chain components VpreB and λ5. Pre-BCR-mediated signals result in clonal expansion of pre-B cells, suppression of apoptosis, targeting of the VDJ-recombination machinery to the *Ig light chain* (*IgL*) loci, and allelic exclusion (14, 15). Analyses of changes in the transcriptome upon tetracycline-controlled pre-BCR induction, uncovered KLF2 as a pre-BCR-induced gene (13). KLF2 expression in pre-B cells was confirmed in KLF2:GFP reporter mice (16). Pre-BCR signals result in Erk5 phosphorylation, which in turn activates the transcription factors Mef2c and Mef2d by phosphorylation. Phosphorylated Mef2c and Mef2d, in turn, activate transcription of the *Klf2* gene and, in parallel, of immediate-early genes, encoding for the transcription factors Jun and Fos, as well as the early growth response proteins Egr1 and 2 that induce pre-B cell expansion (17). In addition, Mef2c/d transcription factors induce IRF-4, a transcription factor important for the termination of pre-B cell expansion and the initiation of immature B cell differentiation (18). Over time, KLF2 accumulates in proliferating pre-B cells and inhibits the Mef2c/d-mediated transcription of the immediate-early genes Jun and Fos and Egr1/2, thus, contributing to the termination of pre-B cell expansion (17). Along this line, ectopic expression of KLF2 resulted in a block of pre-B cell proliferation concurrent with decreased *c-myc* and increased *p21* and *p27* mRNA abundances (19) (**Figure 1**). However, KLF2-deficient mice displayed normal pre-B and immature B cell compartments (16, 20), suggesting that in the absence of KLF2, termination of pre-B cell expansion still occurs and is presumably mediated through *Irf-4* upregulation. As aforementioned, activation of Mef2c/2d by pre-BCR signals results in the upregulation of *Irf-4* expression. Subsequently, IRF-4/IRF-8-mediated upregulation of the transcription factors Aiolos and Ikaros was shown to downregulate pre-BCR expression and to impair cell cycle progression and thereby pre-B cell expansion (21).

As aforementioned, KLF2 expression is induced by the pre-BCR in early B cell development and is maintained in immature B and follicular (Fo) B cells (13, 16, 20, 22). Marginal zone (MZ) B cells show low abundances of KLF2 mRNA and protein whereas B1 cells in the peritoneum display the highest abundance of KLF2 (16, 20, 22). Activation of splenic B cells *in vitro* with LPS, anti-CD40/IL-4 or anti-IgM (anti-BCR) led to decreased KLF2 mRNA and protein abundances (16, 20, 22, 23). In this context, ectopic expression of KLF2 in LPS-activated, proliferating B cells led to an inhibition of B cell activation, expansion, and plasmablast differentiation (19). Therefore, KLF2 acts as a quiescence factor that keeps mature B cells in a resting state. The function of KLF2 as an important quiescence regulator was already postulated in 2000, when KLF2 was found in comparative transcriptome analyses to be highly abundant in resting, naïve B, and anergic B cells, but downregulated in activated B cells (24). In mature B cells, *Klf2* gene expression might be driven by the transcription factor Foxo1 (similar to the Foxo-1-mediated regulation of the *Klf4* gene) as Foxo1-binding sites were found in the *Klf2* promoter and Foxo1-binding to the *Klf2* promoter was described (25). In support, *Klf2* mRNA was reduced in Foxo1-deficient B cells (26). B cell activation results in PI3K-Akt-mediated phosphorylation of Foxo1. Phosphorylated Foxo1 is transported out of the nucleus and becomes transcriptionally inactive (27). Consequently, *Klf2* expression is terminated, which in turn, might enable B cell proliferation and differentiation.

B cells in secondary lymphoid organs can be activated by antigen in either a T cell-dependent (TD) or a T cell-independent (TI) manner. TD activation leads to the formation of a germinal center (GC) reaction in which the BCR of the activated B cell undergoes affinity maturation and Ig class switch recombination occurs. As a result of the GC reaction, B cells with a high affinity BCR either differentiate to memory B cells (Bmem) or to plasma cells (28–31). One study unraveled increased *Klf2* RNA abundances in CD80^+^/PD-L2^+^ Bmem that were shown to quickly differentiate into antibody-secreting cells but did not form new germinal centers (32). Furthermore, single-cell RNAseq of isotype-switched Bmem uncovered a cluster of *Klf2*-expressing Bmem. The cells in this cluster were characterized by low abundances of *Cr2* (CD21), intermediate abundances of *Fcer2a* (CD23), and expressed *Klf2*, *Vimentin-1* and *Prostate androgen-regulated mucin-like protein 1* (*Parm-1*). Based on these characteristics, the authors of this study defined cluster I cells as transitional Bmem (33). Although KLF2 has been detected in Bmem subsets, its functional relevance for Bmem is so far mostly unknown. We speculate that KLF2 in Bmem might functionally contribute to their tissue distribution and retention. In this context, KLF2 expression in Bmem correlated with expression of factors critical for homing and migration, such as Integrin (Itg)β7, Sphingosine-1-phosphate-receptor 1 (S1PR1) and C-C chemokine receptor CCR6 expression (34). Additionally, it is tempting to speculate that KLF2 might keep Bmem in the resting state until they encounter their specific antigen.

GC B cells that differentiate to plasma cells undergo a dramatic morphological change characterized by an increase in cell size and an enlargement of the endoplasmatic reticulum (ER) (31). This process is controlled by a complex regulatory network of transcription factors. Blimp-1 (encoded by the *Prdm-1* gene) is the key transcription factor that drives plasma cell differentiation by promoting Ig production and secretion, and by repressing B ell activation-signature transcription factors Pax5, Bcl-6, Bach2 and the enzyme Activation-induced cytidine deaminase (AID, encoded by the *Aicda* gene) (31). Activated B cells first differentiate into proliferating plasmablasts that are migratory and then into mature, resting plasma cells (35). In plasmablasts in the blood, expression of KLF2 and its target gene *S1pr1* was detected (36). Migration along the sphingosine-1-phosphate (S1P) gradient guides plasmablasts from lymph nodes and spleen to lymph and blood (36). Analysis of KLF2:GFP reporter mice revealed KLF2 expression in IgM and IgA plasmablasts in the blood. In lymphoid organs, the highest frequency of KLF2-positive cells was found within the IgA plasmablast population in mesenteric lymph nodes (mLN), suggesting a pivotal role of KLF2 for IgA plasmablasts and IgA plasma cells (37).

## Functional role of KLF2 in peripheral B cell subsets

The regulatory role of KLF2 in B cell proliferation and activation was primarily analyzed *in vitro* by overexpression approaches and by studying loss-of-function mutants of *KLF2* and their ability to activate NF-κB signaling. Regarding the regulation of quiescence, ectopic expression of KLF2 in pre-B cell cultures and in LPS-activated B cells led to the downregulation of *c-myc* and upregulation of the cell cycle inhibitors *p27* and *p21* (19). Moreover, as shown in monocytes, KLF2 interferes with NF-κB activation (4, 38), a mechanism that might also apply for B cells and B lymphoma cells. Accordingly, *KLF2* loss-of-function mutations as found in human lymphoma cells impaired KLF2-mediated NF-κB suppression in a B lymphoma cell line (11), a topic that will be discussed later in the review article.

To study the functional relevance of KLF2 during B cell development and activation *in vivo*, mouse models with a conditional B cell-specific deletion of a floxed *KLF2* gene were generated. To achieve B cell-specific deletion, either *mb1cre* or *CD19cre* deleter mouse strains were used (16, 20, 22). The B cell-specific deletion of KLF2 resulted in enlarged spleens with an expansion of Fo B cells and MZ B cells (16, 20, 22). KLF2-deficient Fo B cells showed enhanced CD21 surface expression and altered BCR-mediated calcium signals, and thus, as concluded from these parameters and changes in the global gene expression profile partially resembled MZ B cells (16, 20, 22). Fo B and MZ B cells are functionally distinct B cell subsets. Fo B cells migrate between lymphoid organs and give rise to GC upon activation. MZ B cells are a specialized B cell subset located in the splenic marginal zone and their mobility, in contrast to Fo B cells, is limited to shuttling between the marginal zone and the B cell follicle to facilitate antigen transport (39). MZ B cells can develop either from transitional B cells or from follicular B cells (40). Their differentiation is driven by Notch2 signaling. Deletion of *Notch2* or its ligand *Dll-1* resulted in a loss of MZ B cells (41, 42). In an elegant study, induction of Notch2IC (intracellular domain of Notch2 that interacts with DNA-binding protein RBPJ and regulates transcription) resulted in the conversion of Fo B cells to MZ B cells. Upon induction of Notch2IC signaling, *Klf2* (besides *Irf-8* and *Foxo1*) was downregulated (43). These findings are supported by the expansion of MZ B cells observed in KLF2-deficient mice and suggest a role of KLF2 in the cell fate decision and the imprinting of the cellular identity of Fo B versus MZ B cells (**Figure 1**). As described later, loss-of-function mutations of human KLF2 are frequently found in splenic marginal cell lymphoma (SMZL) and play a role in disease onset and/or progression. Immunization experiments showed an increased immune response to TI antigen type 2 (TNP-Ficoll) antigens in B cell-specific *KLF2*-deficient animals compared to controls, which might be due to the observed expansion of MZ B cells and the altered phenotype of KLF2-deficient Fo B cells (22). Immunization with the TD antigen TNP-KLH, however, resulted in reduced antigen-specific IgG titers upon boost immunization. Antigen-specific IgG plasma cells as determined by ELISpot analyses were unaffected in the spleen but were virtually absent in the BM, indicating that loss of KLF2 affects plasmablast homing and/or plasma cell survival in the BM (20).

Importantly, KLF2 deletion profoundly affected mucosal immune responses. KLF2-deficiency resulted in reduction and phenotypic alterations of peritoneal B1 cells (16, 20, 44). Mice with a B cell-specific KLF2 deletion develop fewer and smaller Peyer’s patches (PP) and natural IgA in the serum was reduced (16, 20, 22). Furthermore, B cell-specific deletion of KLF2 resulted in drastically reduced secretory IgA (SIgA) in the gut lumen concomitant with reduced IgA plasma cells in the intestinal lamina propria (LP). IgA plasmablasts and plasma cells, however, accumulated in the mLN and PP, although PP were smaller in size and numbers. Immune responses to immunization with soluble recombinant Flagellin, an immunodominant protein of Salmonella typhimurium, were blunted. In summary, B cell-specific deletion of KLF2 in B cells in mice led to a phenotype similar to that observed in human IgA deficiencies (37).

## KLF2-regulated genes in B cells and plasma cells

**Figure 2:**
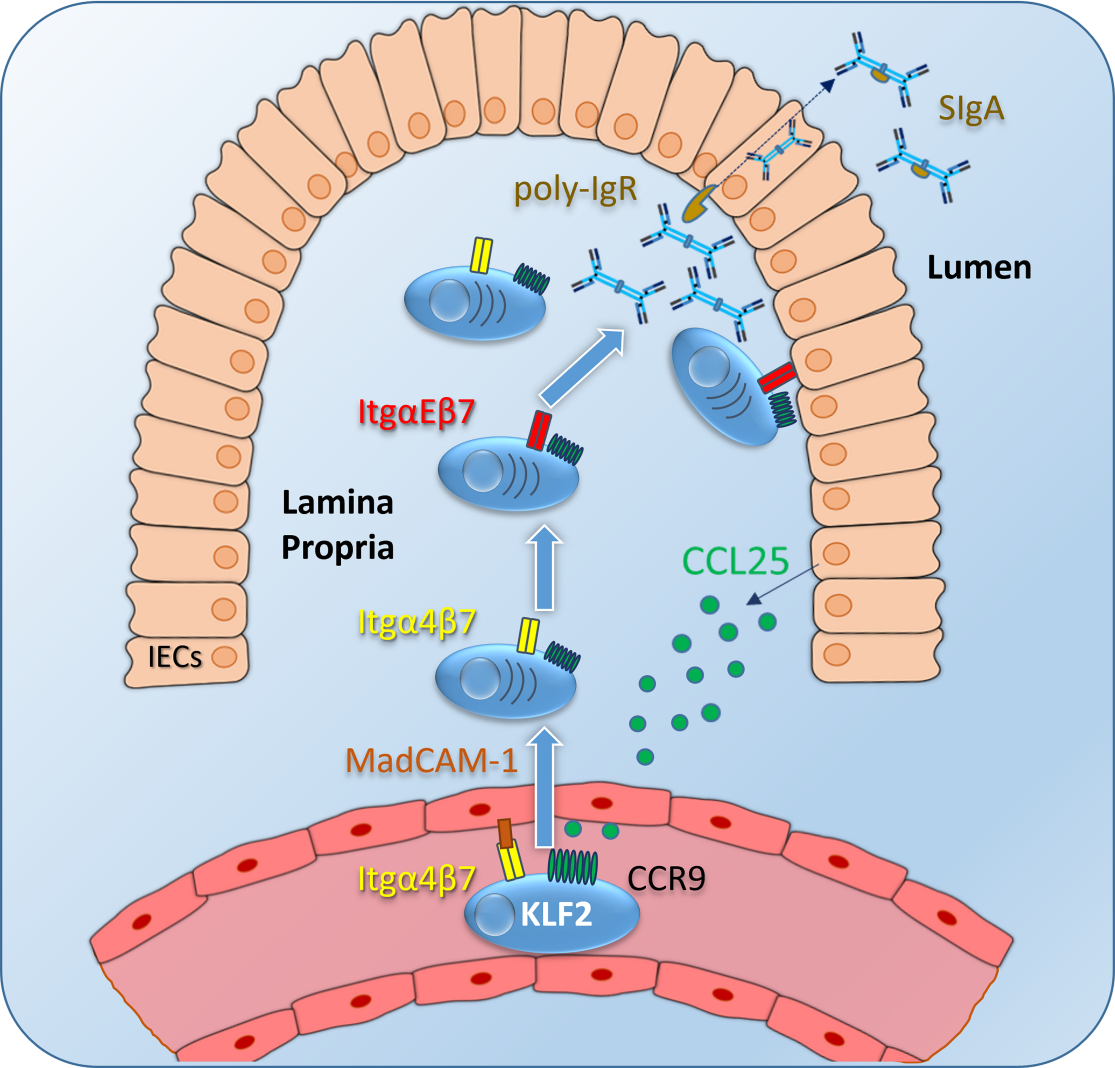
IgA plasmablast homing to the intestinal lamina propria (LP): Intestinal epithelial cells (IECs) express CCL25, which is presented on glucosamine-glycans on endothelial cells of venules as a ligand for the CCR9 receptor on IgA-expressing plasmablasts (and other immune cells). Integrin α4β7 is activated upon CCR9 signaling and binds to its ligand MadCAM-1, followed by plasmablast migration to the intestinal LP. Itgα4β7 and CCR9 expression is induced by KLF2 in IgA plasmablasts. Inside the LP, IgA plasmablasts differentiate into IgA-secreting plasma cells, a subset of those express ItgαEβ7 to localize close to the IECs. This mechanism might facilitate the binding of dimeric IgA to the poly-Ig receptor and might subsequently promote the transcytosis of dimeric IgA through the epithelial layer to the gut lumen. Itg, integrin; SIgA, secretory IgA.

KLF2 acts a major regulator of thymic exit and T cell migration by regulating S1PR1 (45–47). In peripheral murine B cell subsets, one study also described direct binding of KLF2 to the *edg1* promoter (the *edg1* gene encodes for S1PR1) in murine MZ B cells by chromatin immunoprecipitation (ChIP) (22). In this study, *S1pr1* mRNA was shown to be downregulated in KLF2-deficient MZ B cells and upregulated in Fo-deficient B cells despite the lack of KLF2 binding to the *edg1* promoter in Fo B cells (22). Two other independent studies demonstrated that S1PR1 mRNA and protein were not significantly altered in KLF2-deficient Fo B cells (16, 20). Therefore, the involvement of KLF2 in the regulation of *S1pr1* expression in MZ B cells and Fo B cells remains unresolved. In IgA plasmablasts, however, RNASeq data confirmed the KLF2-dependent regulation of *S1pr1* and *S1pr4* mRNAs, which were both significantly reduced in KLF2-deficient IgA plasmablasts in the mLN (37). Therefore, KLF2-mediated regulation of S1PRs might contribute to plasmablast migration and homing to the bone marrow as well as mucosal effector sides (**Figures 1**, **2**).

The chemokine receptor CXCR5 recognizes the chemokine CXCL13 and is important for the positioning of B and T cells inside the B cell follicles in lymph nodes and the spleen (48) and for the shuttling of MZ B cells between the follicle and the marginal zone of the spleen (39). In T follicular helper (TFH) cells, KLF2 binds directly to the *Cxcr5* promoter (as shown by ChIP) and represses *Cxcr5* expression. Downregulation of KLF2 caused by ICOS signals *via* Foxo1 resulted in *Cxcr5* upregulation that is critical for TFH-positioning in the B cell follicle (49). In contrast to the well described regulation in TFH cells, KLF2-mediated regulation of *Cxcr5* in B cells remains controversial: one study described downregulation of CXCR5 mRNA and protein in KLF2-deficient MZ B cells and an upregulation in KLF2-deficient Fo B cells (22). However, two other studies were not able to confirm this regulation (16, 20). Therefore, it remains unclear whether KLF2 might be involved in the regulation of MZ B cell-shuttling between the marginal zone and the follicle, or in Fo B cell-positioning within the follicle as shown for TFH cells. Hence, resolving the role of KLF2 in MZ B-shuttling and Fo B cell migration within the follicle will require more sophisticated spatial and temporal analyses.

Genome-wide microarray RNA expression analyses in Fo B cells in two different mouse strains with a B cell-specific *Klf2* deletion (either *CD19Cre*- or *mb1Cre*-mediated) identified the surface receptors L-Selectin (CD62L) and Integrin (Itg) β7, which are important for migration and homing, as KLF2-regulated factors (16, 20) (**Figure 1**). While L-Selectin as a major factor of leucocyte extravasation, plays an important role in B cell migration from blood to lymph nodes, Itgβ7 is known for its specific role in mucosal lymphocyte migration. It was demonstrated by chromatin immunoprecipitation (ChIP) that KLF2 directly binds to the *Itgβ7* promoter in B cell lines (50). On protein level, loss of surface L-Selectin and surface Itgα4β7 was demonstrated in KLF2-deficient splenic Fo B cells and B cells in the blood (16, 20). Moreover, in KLF2-deficient TACI^+^/CD138^+^ IgA plasmablasts, Itgα4β7 was downregulated (37). As the Itgα4 chain was virtually absent on the surface of KLF2-deficient IgA plasmablasts, not only Itgα4β7 but also surface expression of Itgα4β1, which is critical for BM homing, is impaired (37). Besides downregulation of L-Selectin and Itgβ7, a significant reduction of *S1pr4* and an increase of *S1pr3* transcripts in KLF2-deficient Fo B cells was detected (16). While S1PR3 plays a role for MZ B cell positioning but is dispensable for lymph node motility, the function of S1PR4 in B cells is unclear (39, 51).

KLF2 directly induces Blimp1 during Th1 cell differentiation by binding to the *Prdm1* promoter (52) but it remains unclear whether Blimp1 is also controlled by KLF2 during plasma cell differentiation. Based on the findings that KLF2-deficent mice had reduced numbers of antigen-specific IgG-secreting plasma cells in the BM and that natural IgA was reduced in their serum, the effect of KLF2 deletion on plasmablast and plasma cells subsets was thoroughly assessed by our group (20, 37). Plasmablasts were defined as CD19^+^/B220^+^/TACI^+^/CD138^+^ cells with a high frequency of proliferating Ki67^+^ cells, whereas plasma cells were identified as CD19^lo/neg^/B220^-^/TACI^+^/CD138^+^ which are non-proliferating (35, 37). Analysis of plasma cell compartments in B cell-specific KLF2-deficient mice revealed a severe dysregulation of the compartmentalization of IgA plasmablasts and IgA plasma cells. In these mice, IgA plasmablasts and IgA plasma cells were virtually absent in the BM, reduced in the blood, the spleen and importantly, the intestinal LP. However, IgA plasmablasts and IgA plasma cells accumulated in mLN of KLF2-deficient mice (37). RNAseq as well as flow cytometric analyses of KLF2-deficient IgA plasmablasts compared to controls identified L-Selectin, Itgβ7, ItgαM, and chemokine receptor CCR9 as KLF2-regulated factors (37). Surface CCR9 on IgA plasmablasts was significantly reduced concomitant with an impaired migration towards a CCL25 gradient *in vitro* (37). Together, reductions of Itgβ7 and CCR9 expression in KLF2-deficient IgA plasmablasts led to compromised IgA responses caused by impaired migration from mLN to the LP of the small intestine and colon (37). Hence, KLF2 regulates the expression of the important gut-associated lymphoid tissue (GALT)-homing factors Itgβ7 and CCR9 (**Figures 1**, **2**). Upon KLF2-regulated expression of CCR9 and Itgβ7, IgA plasmablasts are attracted to the LP by gradients of CCL25, the ligand of CCR9. CCL25 is secreted by e.g., intestinal epithelial cells (IEC) (53). CCL25-binding to CCR9 activates Itgα4β7 that binds to MadCAM-1 on endothelial cells and leads to the extravasation of plasmablasts to the mucosal LP (54, 55). Inside the intestinal LP, IgA plasmablasts differentiate to mature IgA plasma cells. A subset of those express ItgαEβ7 which enables them to bind to E-Cadherin on IECs, a mechanism that might promote dimeric IgA binding to the poly-IgR and facilitate transcytosis of dimeric IgA to the gut lumen [(56), **Figure 2**].

In addition to the regulation of Itgα4β7, the expression of the ItgαM chain was also affected in KLF2-deficient IgA plasmablasts. ItgαM is a binding partner of Itgβ2, which is important for lymph node egress of B cells (57). Moreover, ItgαM was absent on KLF2-deficient IgA plasmablasts compared to their wildtype counterparts (37). The dysregulation of ItgαM together with the aforementioned reduction of S1PR1 might be the cause for the observed accumulation of IgA plasmablasts/plasma cells in the mLN and in the remaining PP of KLF2-deficient mice (37). Hence, KLF2 might be involved in the process of lymph node exit of IgA plasmablasts presumably by regulating ItgαMβ2 and S1PR1.

In summary, KLF2 contributes to the control of the quiescent, resting state of mature B cells and pre-B cells by controlling cell cycle regulators (c-myc, p21, and p27) and immediate-early transcription factors (such as Jun, Fos, and Egr1/2), respectively. Moreover, KLF2-regulated genes are crucial for migration and homing of naïve B cells, activated B cells, and plasmablasts. KLF2-regulated gene products include integrins (Itgα4β7, Itgα4β1, and ItgαM), selectins (L-Selectin), and chemokine receptors (CCR9) as well as Sphingosin-1-phosphat-receptors (S1PR1, S1PR3, and S1PR4) in IgA plasmablasts. By regulating the expression of these factors, KLF2 controls the exit of IgA plasmablasts from the lymph node as well as their homing to the intestinal LP.

## KLF2 in B cell-related diseases and malignancies

### Splenic marginal zone lymphoma

In humans, splenic marginal zone lymphoma (SMZL) is a low-grade B cell lymphoma, with variable clinical course. Clinical diagnosis is rather difficult as specific phenotypic and genetic markers are lacking. In approximately one third of SMZL cases, the IgHV1-2 heavy chain that harbors few somatic mutations and a long CDR3 region is expressed (58, 59) and approximately one third of SMZL cases harbor a hemizygous deletion of chromosome 7q with a so far unsolved role in the pathogenesis of SMZL (60–62). Transcriptome and mutational analyses have revealed candidate genes that may contribute to disease onset and/or progression. Mutations were predominantly detected in the *KLF2* and the *NOTCH2* genes. KLF2 was inactivated by mutations in 42% of SMZL patients/cases (11). This is in line with findings that KLF2-deficient mice display a strong expansion of MZ B cells (16, 20, 22). Based on the mutations found in SMZL patients, expression constructs with genes encoding for different KLF2 mutant forms were generated. The effect of these KLF2 mutants on NF-κB activation was assessed in *in vitro* reporter assays in HEK293T cells and OCI-LY19 B-lymphoma cells. KLF2 mutants failed to suppress NF-κB activation in contrast to non-mutated KLF2 (11). Constitutive activation of the NF-κB signaling pathway contributes to SMZL pathogenesis by promoting MZ B cell survival and expansion (63, 64).

### Multiple myeloma

The hallmark of Multiple Myeloma (MM), a malignant disease, is the expansion of plasma cells. Clinical signs include hypercalcemia, renal failure, anemia, and bone lesions. Moreover, MM is characterized by plasma cell expansion in the BM and the presence of free IgL chains, the so-called Bence Jones proteins that can be found in the serum and the urine of MM patients (65). Genetic predispositions such as mutations in the *N-RAS*, *K-RAS* or *EGR1* genes as well as translocations are primary events in the onset of MM (65, 66). Deregulation of histone methylation can also contribute to MM. In this context, the chromosomal translocation t (4,14) (p16;q32) can be found in up to 20% of MM patients. This translocation results in the overexpression of WHSC1, a histone H3 lysine 36 (H3K36) methyltransferase (67). Furthermore, the KDM3a histone demethylase that catalyzes the removal of H3K9 mono- and di-methylations, is expressed in MM lines and was shown to be essential for MM cell proliferation and survival. *KLF2* was identified as a target gene of KDM3a. KLF2 is highly expressed in MM cell lines (68). Downregulation of KLF2 resulted in an impairment of MM cell proliferation and in the induction of apoptosis. *IRF-4* was identified a KLF2-regulated gene in MM cell lines. Together, KDM3a, KLF2, and IRF-4 regulate the expression of *ITGβ7*, an essential integrin for MM homing to and adhesion in the BM (69). As aforementioned, ITGβ7 is a crucially important KLF2-regulated target gene in healthy B cells and plasma cells. Therefore, KLF2 is involved in MM cell adhesion and BM homing. Moreover, KLF2 is involved in the regulation of the angiogenic factors EGFL7 and ITGβ3 in MM cells. KLF2 expression was increased by ITGβ3 signaling which in turn led to upregulation of EGFL7, thereby enhancing MM cell expansion (70). In contrast to naïve B cells, MM cells proliferate in the presence of KLF2. As aforementioned, KLF2 in MM cells promotes their proliferation and survival. Therefore, the complex interplay of the various signaling pathways implicated in the pathogenesis of MM (i.e., the RAS/RAF/MEK/ERK, the PI3K/AKT, the JAK/STAT, and the NF-κB pathways (71) with the KLF2 signaling network in MM cells needs to be further investigated.

### IgA deficiencies

As aforementioned, B cell-specific deletion of *Klf2* in the mouse resulted in a profound disturbance of the localization of IgA plasma cells concurrent with the absence of SIgA in the gut lumen and feces (37). These phenotypes are strikingly similar to those found in human IgA deficiencies (72). Loss of Itgβ7, a central player of IgA plasmablast/plasma cell homeostasis, is implicated in the human Kabuki syndrome. In a corresponding mouse model, deletion of the gene encoding for the Kmt2d histone methyltransferase led to a decrease of Itgβ7 expression, which consequently resulted in a defective homing of IgA plasmablasts to the gut (73). As *Itgβ7* is also a direct target gene of KLF2, it will be of great interest to study the effect of KLF2 loss-of-function mutations on the onset and progression of gut-related diseases, such as Ulcerative colitis and Crohn’s disease.

### B cell abnormalities

Recently, a novel mutation in the human *KLF2* gene was discovered that leads to the disruption of the highly conserved zinc finger domain required for the nuclear transport and DNA-binding. The patients showed lymphopenia with decreased B cell numbers, lower numbers of switched memory B cells, and reduced serum IgG1. Moreover, L-Selectin on blood B cells was downregulated. In addition, this mutation also resulted in an imbalance of various T cell subsets (74).

## Future perspectives

KLF2 is a central regulator of not only B cell and plasma cell differentiation, activation, and migration, but is equivalently important in other immune cells. KLF2 alterations have been associated with a multitude of diseases, such as adipogenesis, atherosclerosis, thrombosis, asthma, arthritis (3, 4, 7–9, 12). Thus, the challenge for further studies will be the identification and characterization of the KLF2-regulated signalosome, transcriptome, and proteome in various cell types in immune responses and diseases.

## Author contributions

JW and WS conceptualized and wrote the manuscript. All authors contributed to the article and approved the submitted version.
